# A Framework for Multi-Agent UAV Exploration and Target-Finding in GPS-Denied and Partially Observable Environments

**DOI:** 10.3390/s20174739

**Published:** 2020-08-21

**Authors:** Ory Walker, Fernando Vanegas, Felipe Gonzalez

**Affiliations:** Queensland University of Technology, Brisbane City, QLD 4000, Australia; f.vanegasalvarez@qut.edu.au (F.V.); felipe.gonzalez@qut.edu.au (F.G.)

**Keywords:** POMDP, Deep Reinforcement-Learning, UAV, multi-agent, search

## Abstract

The problem of multi-agent remote sensing for the purposes of finding survivors or surveying points of interest in GPS-denied and partially observable environments remains a challenge. This paper presents a framework for multi-agent target-finding using a combination of online POMDP based planning and Deep Reinforcement Learning based control. The framework is implemented considering planning and control as two separate problems. The planning problem is defined as a decentralised multi-agent graph search problem and is solved using a modern online POMDP solver. The control problem is defined as a local continuous-environment exploration problem and is solved using modern Deep Reinforcement Learning techniques. The proposed framework combines the solution to both of these problems and testing shows that it enables multiple agents to find a target within large, simulated test environments in the presence of unknown obstacles and obstructions. The proposed approach could also be extended or adapted to a number of time sensitive remote-sensing problems, from searching for multiple survivors during a disaster to surveying points of interest in a hazardous environment by adjusting the individual model definitions.

## 1. Introduction

In recent years the use of Unmanned Aerial Vehicles (UAVs) has been broadly explored for a number of applications, both in consumer and industrial operation environments. Many tasks require the capacity for autonomous searching or surveying of a known or unknown environment in the presence of hazards. Such applications include the broad field of search and action tasks, from search and rescue [1], environmental sampling and data collection [2,3,4], pursuit of targets in complex environments [5,6], even underground mining and surveying of confined spaces [7].

Often-times exploration tasks are time-sensitive and require the use of multiple agents to carry out the mission objective. However, coordinating multiple UAV agents over complex environments in the presence of partially observable obstacles remains a challenge. There are multiple works involving searching or navigating an unknown environment using single and multiple agent configurations [8]. Solutions in the past have not allowed for operation of the UAV under uncertainty [9] or operation in continuous action spaces, using a discrete list of actions to control the operation of the UAV [10].

Modelling of many robotic control problems involving uncertainty is usually performed with the Partially Observable Markov Decision Process (POMDP) [11] framework. The main reason being that POMDPs are useful for modelling uncertainty within a system. In the past POMDPs were most effectively solved by classical algorithms and solvers [12,13,14]. However, these approaches have a number of limitations, including discrete action spaces [12] or difficulty modelling continuous action spaces [12,13], pauses in operation to calculate the next optimal trajectory [12,13] in the case of online planners, or long pre-planning times for each new environment. While performance of traditional solvers is excellent for solving discrete time-independent planning problems [15], their limitations could be detrimental when applied to low-level continuous control.

In recent years however there has been an explosion in the application of machine learning techniques to solving POMDP tasks as a result of advancements made to deep-reinforcement learning techniques. They have shown that they are exceptional when solving well defined MDP and POMDP control tasks, from simulated robotic control tasks [16,17], to atari video games [18], with deep reinforcement learning based approaches even out-performing human experts in the games of Go [19,20], Dota 2 [21] and Starcraft 2 [22].

Included in the tasks that have seen an increase in the use of machine learning techniques are UAV control tasks [23]. Recently a deep Q-network approach was applied to the exploration and navigation of outdoor environments [24], and a multi-agent deep deterministic policy gradient method was applied to multi-agent target assignment and path planning in the presence of threat areas [25]. However, the application of such techniques to control the exploration of multiple UAV agents in partially observable and hazardous environments remains unrepresented.

This paper maintains a focus on the problem of target-finding using multiple UAV agents in partially observable environments.The solution presented in this paper can be expanded and adapted as necessary to a number of remote sensing problem spaces, from searching for survivors in a variety of environments such as disaster zones, buildings, caves systems, and open or forested areas, to surveying potentially hazardous or difficult to reach points of interest. We present the multi-agent UAV target-finding problem as two problems; a decentralised multi-agent planning problem, and a local control problem. The UAVs must be capable of searching local continuous environments containing unknown hazards, while also being directed between local environments within the global environment.

This paper presents a multi-agent target-finding framework, using the Robotic Operation System 2 (ROS2) platform [26], to search partially observable occupancy map style environments using multiple simulated UAV agents. The Adaptive Belief Tree (ABT) solver [12] is used for validating the planning component of the framework, while a Proximal Policy Optimization (PPO2) [27] algorithm is used for producing a solution for and validating the local control component of the framework.

The main contribution of this paper is a hierarchical framework that combines decentralised POMDP based planning and deep reinforcement learning to enable multiple UAV agents to search GPS-denied, partially observable occupancy map style environments for a target while adapting to unknown obstacles in real-time. Secondary contributions include modelling of both the multi-agent planning problem for use with the TAPIR POMDP software package and the local control model in the form of an open-ai gym environment that uses occupancy-grid style maps.

## 2. Background

The POMDP framework is suitable for modelling sequential decision making for robotic agents operating in the real world, as robotic agents rarely if ever have access to measurements and sensing free from uncertainty.

Formally, the POMDP framework can be defined by an 8-tuple (S, A, T, O, Ω, R, b0, γ). Where:

- S is a set of states,
- A is a set of potential actions,
- T is the transition function, defining the probability of transition between states,
- O is a set of observations, Ω is the observation function, defining the probability of observing *o* from state *s* after taking action *a*,
- R is the reward function,
- b_0_ is the initial belief,
- and γ is the discount factor.

An agent (UAV) in a POMDP formulation does not have knowledge of the true state of the environment. The agent instead maintains a continuous belief *b(s)* over the potential states. It updates this belief after taking an action *a* and making an observation *o*. Consider this belief a distribution of all possible states for the agent given the transition and observation functions T and Ω, with less certain functions (modelled for less certain problems) resulting in a larger belief space, and more uncertainty in the true state of the agent and environment.

The objective of a POMDP solver is to identify a sequence of actions that maximize the total expected discounted return of the model. Rather than calculating for a known true state *s* however, the POMDP solver uses an initial belief of potential states b0.

The solution a POMDP solver provides is a policy π that maps belief space to action space and identifies which action will maximize the expected discounted reward given a belief b(s). The optimal policy is then represented by π* which yields the maximum reward value for each belief state, which is denoted by V*. V* (b) is therefore the value function that accounts for the maximum reward value for any given belief *b*.

Adaptive Belief Tree (ABT) [12] is a recent online POMDP solving alogrithm implemented in the TAPIR software toolkit [28] capable of solving complex POMDP problems with significant state-spaces despite potential changes in the problem space by efficiently adapting previously generated policies during run-time to incorporate new information and produce near-optimal solutions.

In this work the ABT solver is applied to solving the global planning problem modelled as a POMDP. Further work would extend this problem model to include dynamic responses to a changing environment and planning in a completely unknown environment.

Solving the POMDP problem of local UAV exploration control involves optimizing a policy with respect to a future discounted reward. The primary goal of deep reinforcement learning within our framework is to learn a near-optimal policy for the local exploration control problem. Deep Reinforcement Learning leverages the properties of deep neural networks and reinforcement learning algorithms to produce policies that maximise a reward function for potentially complex problems. For validation of the framework we opted to use the Proximal Policy Optimization (PPO2) algorithm that is implemented in the Stable Baselines [29] project. However, the framework is designed in a modular fashion which permits the use of alternative algorithms to solve the problem.

A key benefit of applying Deep Reinforcement Learning to the problem is the reduced control overhead required when implementing a learned policy and the near-instantaneous response to new information available to the UAV at any given time, without the need for costly re-calculation as more information is acquired about the environment. The PPO2 algorithm has been shown to learn complex policies for a variety of continuous control tasks, and is well suited to the control task within this framework.

## 3. Problem Definition

**Figure 1:**
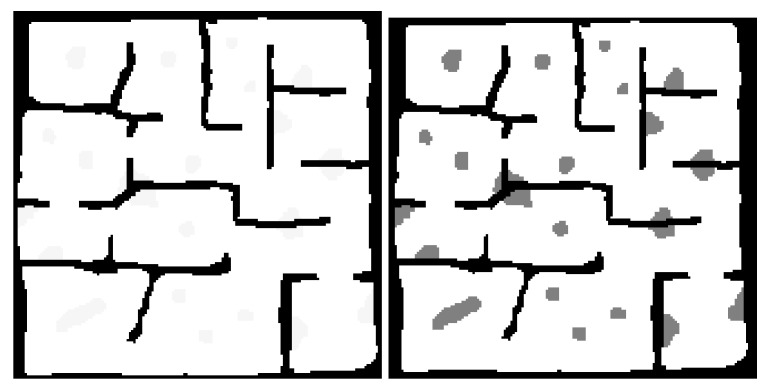
Prior Knowledge of the Environment vs. True State of the Environment.

The problem that the proposed framework aims to solve is that of searching a gps-denied, partially observable environment for a target using multiple UAV agents. The problem assumes the following:

- A rough map of the environment is known prior to operation. This map helps define the connections between the search regions of the environment. Such a map might exist in the form of a floor-plan for a building or a pre-existing map for a cave system.
- Obstacles, obstructions and minor changes to the shape of the local environments are unknown to the agents prior to operation. Figure 1 shows the difference between the known and unknown environment information.
- Agents are capable of some form of Simultaneous Localisation and Mapping (SLAM).
- Local agent localisation is perfect or near-perfect.
- The target does not move.
- There is an equal chance of the target being at any location within the environment.
- The search occurs on a single 2D-plane—i.e., the agent doesn’t benefit from changing its altitude during the search as the obstacles are floor to ceiling.

The implementation presented in this paper assumes that UAV agents are equipped with a front facing sensor (a camera) with a range of seven (7) metres and a horizontal field of view of ninety (90) degrees. In practice, the kind of sensor the UAV is equipped with only matters during training of the control policy outlined in Section 6. During operation the Planning and Control (PAC) framework proposed only requires that the SLAM system used by an agent outputs a grid-map of the environment and the agent’s pose and velocity within that grid map. The decoupling of SLAM from the PAC framework means that it can readily be expanded for platforms using other sensors and implementations of SLAM. This also means that the sensor dynamics and sensor noise are not considered by the PAC Framework during operation. A simplified approximation of a SLAM system is simulated in order to validate the PAC framework.

## 4. Framework Definition

Developing the framework required the problem to be broken down into two main problem components:

- Planning for multiple agents over potentially large maps to find a target with an unknown location.
- Individual agent exploration control within local continuous partially observable environments.

**Figure 2:**
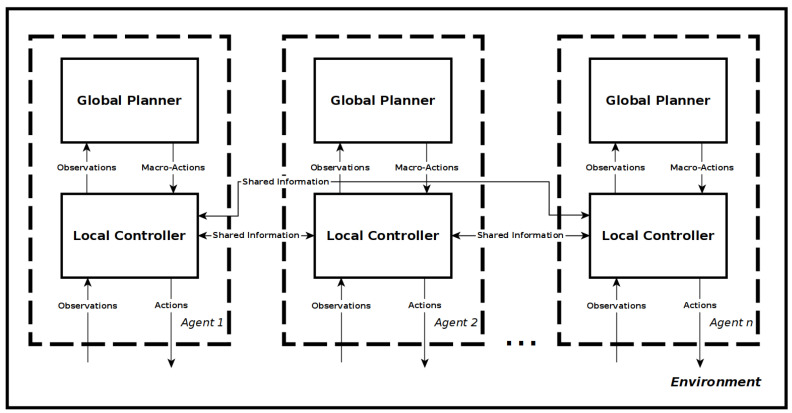
Framework Architecture.

To enable the framework to scale to large map sizes while also enabling local control, it was decided that the framework would use occupancy grid maps with a high level graph-map definition of the environment. The local controller would use local mapping and position information to control the agent, while the global planner considered the environment as a discrete graph map. The information contained within the map and how an environment is defined are outlined further in Section 4.1. It was decided for the global planner to use a decentralised approach, with each agent having its own planner and communicating the relevant information as necessary to all other agents. Each agent also has a local controller that handles communication between agents, receives macro-actions from the global planner, processes the pose, velocity and mapping information into an abstraction that can be used by the control policy, and outputs actions from the control policy to the flight controller. Figure 2 shows the resulting two layer-framework architecture for n-agents.

### 4.1. Environment Definition

Proper definition of the environment and the information available to the agent is instrumental to the problem definition and by extension the operation of the proposed framework. The environments used by this framework are occupancy style grid-maps and are composed of four main types of cells:

- Obstacles known prior to operation. Defined as a value of −3 in the occupancy-map.
- Obstacles unknown prior to operation. Defined as a value of −2 in the occupancy-map.
- Explored empty space. Defined as a value of −1 in the occupancy-map.
- Unexplored empty space. Defined as a value of >= 0 in the occupancy-map.

**Figure 3:**
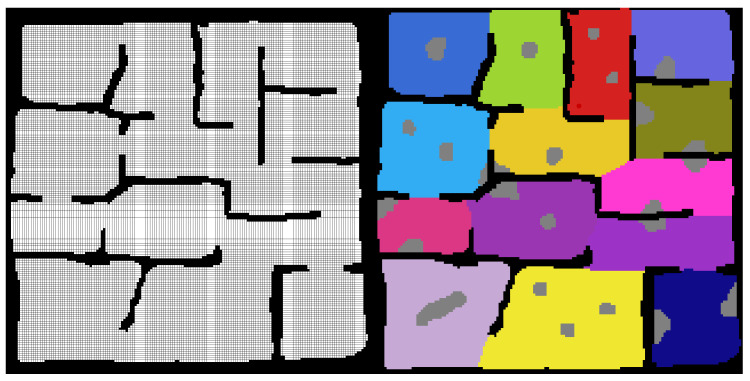
Map Creation: Defining the Known Obstacles, Search Regions and Unknown Obstacles. **Black Cells**: Known Obstacle Cells, **White Cells**: Explored Empty Cells, **Grey Cells**: Unknown Obstacle Cells, **Coloured Cells**: Unexplored Empty Cells (Search Regions).

The map is further broken down at a higher level into search regions. These are differentiated via the use of different positive integers within the occupancy-map and their shape and size is generally defined by the natural borders created by known obstacles. The known obstacles are assumed prior knowledge within the problem space, and are defined from a pre-existing floorplan or map if one exists. Figure 3 shows the first steps in the creation of one of the test maps that were used for validation of the framework. First the known obstacles are defined, and then the search regions are filled into the empty space and unknown obstacles are added. The test map shown has existing known structures as a building or man made structure might have.

**Figure 4:**
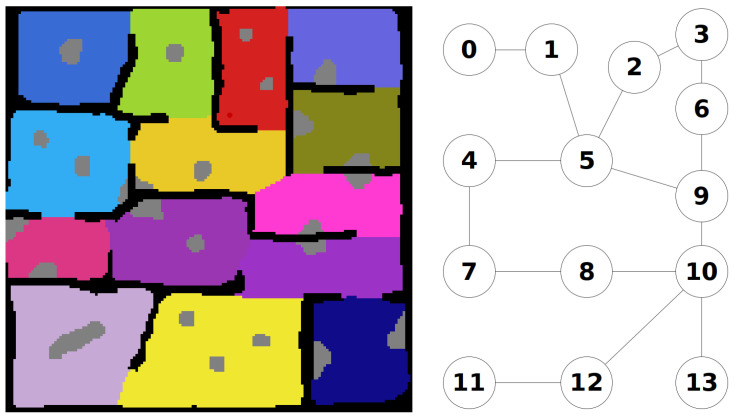
Graph Map Definition From Search Regions. **Black Cells**: Known Obstacle Cells, **White Cells**: Explored Empty Cells, **Grey Cells**: Unknown Obstacle Cells, **Coloured Cells**: Unexplored Empty Cells (Search Regions).

Once the regions are defined, the connections between the regions can be used to define a graph map of the environment. An example of this is shown Figure 4.

**Figure 5:**
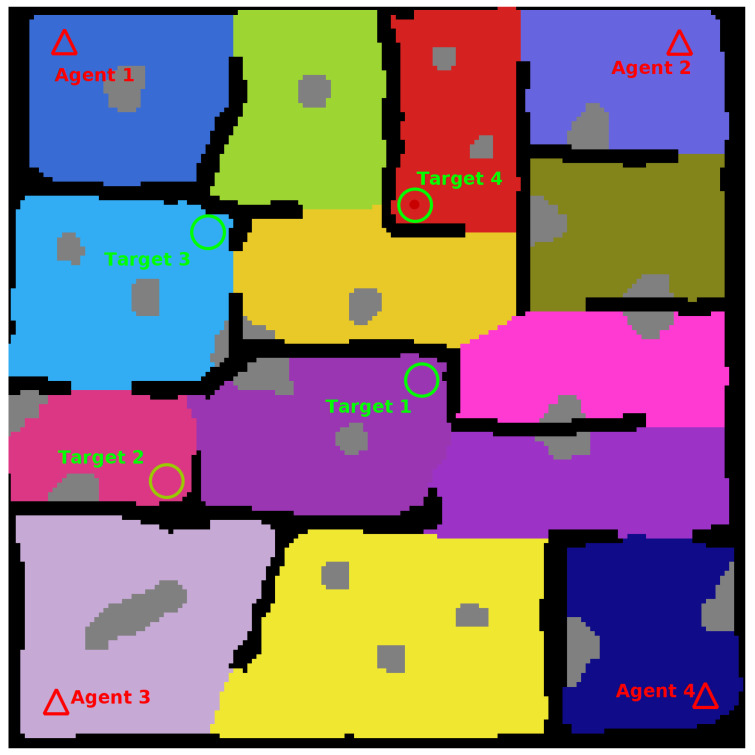
Complete Map. **Black Cells**: Known Obstacle Cells, **White Cells**: Explored Empty Cells, **Coloured Cells**: Unexplored Empty Cells (Search Regions), **Grey Cells**: Unknown Obstacle Cells, **Triangles**: Agent Spawn Locations, **Circles**: Target Spawn Locations.

To finish creation of the map locations for the agents and targets to spawn are added. A complete map is shown in Figure 5.

## 5. Decentralised Multi-Agent Planner

Within the framework, each agent has their own global planner that is responsible for generating macro-actions for that agent. These macro-actions direct the UAV toward connected nodes within the graph-map according to a policy generated by the planner. The goal is to find a target within the environment in as few moves as possible. In its simplest form the planner directs the agent to search the graph-map of the environment until the agent is found or the environment is completely searched. For this paper the planner operates under the assumption that the target distribution is uniform across the environment. However, it would be simple to add the functionality for weighted distributions in the future. The positions of all agents are considered fully observable for the duration of the task, as localisation and communication are assumed, however the partially observable target location means that the problem must be modelled as a POMDP.

The policy for the POMDP model is generated using the online POMDP solver platform TAPIR, using the ABT algorithm. The implementation allows for the definiton of a move action for all nodes, however only those actions that are legal are considered during rollout, i.e., only actions that would result in the UAV moving to a connected node from its current node are considered during planning. The model also assumes that any node that the UAV visits will be searched and a penalty is given to an agent for occupying the same space as another agent in order to reduce the likelihood of the planners trying to have two agents occupy the same node, as this functionality is not available in the local controller at this time.

Due to the decentralised nature of the approach, coordination between agents is achieved in a loose fashion. Each agent is aware of the location of all other agents. During rollout each planner considers locations occupied by other agents as searched, just as it would consider a location occupied by its own agent. A planner also assumes during policy generation that other agents are equally likely to move to new locations at each time step, subsequently searching those locations. This causes the generation of a variety of future beliefs during rollout which collapse into a single belief whenever the positions of each agent are observed. By building these assumptions into the model, the planners produce individual agent policies that attempt to maximize a shared reward by finding the target in as few steps as possible, enabling decentralised collaboration. For instance, agents won’t navigate toward regions occupied by other agents, as they assume that those areas will be searched by the agents occupying those regions. The problem is formulated as a Decentralised POMDP, considering only the actions for a single agent based on information received from all agents. It is modelled as shown in Table 1.

## 6. Local Control Policy

The local controller component of the framework is composed of a single deep reinforcement learning control policy to generate actions and the ancillary components required for communication and observation generation for the local control policy and global planner. The following section details how the local control policy is generated. The goal of the Local Control Policy is to generate actions from observations such that the UAV navigates a local continuous portion of the global environment, exploring unexplored areas and avoiding obstacles as it discovers them. The actions that the policy generates are velocity commands to increase or decrease the velocity of the agent in the x, y, and yaw axes. To generate this control policy, a custom OpenAI gym environment was created, and then an agent was trained on that environment using the PPO2 implementation within Stable Baselines.

### 6.1. Open AI Gym Environment Definition

The Open AI Gym environment used to train the agent needed to be a good proxy for the simulated operation of an agent within the framework. Ideally it also needed to be lightweight in order to increase the speed at which the agent was trained. Because the agent was required to search environments with unknown obstacles, the problem also needed to be modelled as a POMDP. The problem was modelled as shown in Table 2.

Definition of the environment aspect of the state space for the problem was relatively simple. The gym environment uses one or more occupancy-maps, as defined in Section 4.1, as the training environments for the agent, each with only a single search region and no graph-map, as those features are only required for the global planner aspect of the framework. The map changes as the UAV explores, replacing unexplored cells with explored cells according to simulated sensing. The UAV location is simply the agents x, y and yaw value within the map, and is updated according to the agents velocity at the beginning of each time step.

The action space for the agent is a continuous space between −1 and 1 for each of the control axes of x, y, and yaw. The policy outputs a vector [x, y, yaw] at each time step that changes the agents velocity.

**Figure 6:**
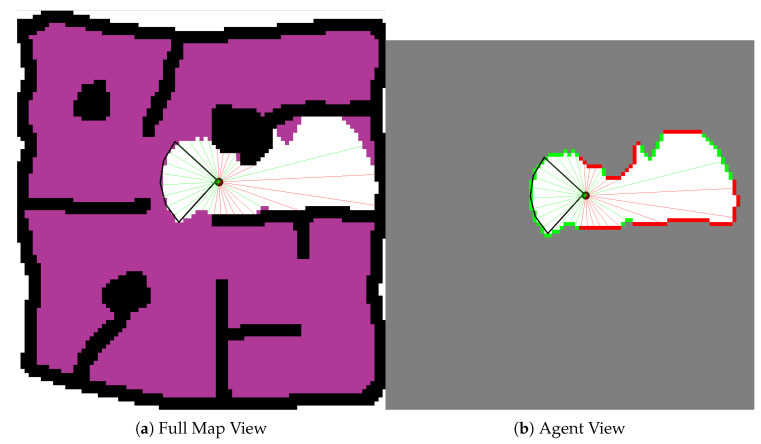
Full Map View vs. Agent View. **Black Cells**: Obstacle Cells, **White Cells**: Explored Empty Space Cells, **Purple Cells**: Unexplored Empty Cells, **Grey Cells**: Unexplored Area, **Red Cells**: Observed Obstacle Cells, **Green Cells**: Safe Edges of Unexplored Region, **Black Cone**: Field of View of Agent’s Sensor, **Red Lines**: Obstacle Distance Readings, **Green Lines**: Safe Distance Readings, **Circle**: UAV.

With respect to the observation space, the agent’s velocity is fully observable. However the control policy can only receive information about the explored environment, and it does so via the use of thirty-two (32) distance readings and a corresponding type index. These readings are an abstraction of the UAVs pose within the local environment and the state of that local environment. They are generated irrespective of the sensors used by the agents. At each time step according to the agents pose within the environment, thirty two (32) distance readings spaced equally around the agent are projected to the first unexplored cell or obstacle in line with that reading. The type index is then updated to reflect whether the distance reading indicates an obstacle at that location or not. Figure 6 shows the agent within an environment, the cone of vision of the assumed front-facing sensor and the distance readings that are generated at each time step according the the UAV’s pose and knowledge of the environment.

The environment is simulated at 10 hz i.e., observations are made and actions are generated every 0.1 s.

### 6.2. Defining The Simulated Agent

The above Open Ai Gym training environment can be used to produce a control policy for different kinds of agents by changing the model of the agent within the environment. As a result the gym environment developed could be used to train a large sluggish UAV equipped with a LIDAR sensor, or a small agile UAV equipped with a front facing camera. In this case, a generic simulated agent with the characteristics shown in Table 3 was trained in the environment. Note that the characteristics listed are not the extent to which an agent might be modelled, and are only what were considered necessary for the generic agent model.

The radius defines the size of a UAV and how close it can come to obstacles without triggering a collision and incurring a penalty.

The Linear and Angular Velocity Limits denote the max possible velocity in the linear and angular axes of control (x, y and yaw). The Response Type characteristic is where the simulated agent deviates largely from real world UAVs. In this case the simulated agent is modelled using a Linear Response. For instance, if the simulated agent has a velocity of [0, 0, 0] and receives a control vector of [1, 0, 0], after one time step the agents velocity will be [1, 0, 0] with the average velocity of the agent over that time step being [0.5, 0, 0]. The Linear and Angular Action Scale parameters define the maximum change in velocity that can be requested by the controller at any time, i.e., the largest velocity delta for the agent over a single time step is capped at [0.25 m/s, 0.25 m/s, 0.125 ω/s]. Essentially they are a gain applied to the actions produced by the policy, which have a continuous range of [−1, 1] for each axis. The values of these parameters were selected because they seemed reasonable for the desired simulated agent.

The sensor type of the simulated agent was selected to be a front facing camera with a Sensor Range of seven (7) metres, and a horizontal field of view (FOV) of 90${}^{\circ }$. This sensor type was then modelled into the environment so that the agent could make observations during training, and the policy could learn how to search using that sensor. Training the agent is the only time when a model of the sensors used for making observations are required by the framework. The training step uses the model, which can be an approximation, of the sensor to simulate mapping of environments, such that the control policy learns the most optimal way to search unknown environments using that sensor while avoiding collisions. For instance, by simulating a LIDAR sensor, instead of a front facing camera, the control policy produced would be optimized for use with LIDAR based systems, instead of a system with a limited front-facing sensor.

Changing any of these parameters and how the agent is defined and modelled within the environment would change the learnt policy accordingly and enable control of a variety of UAV platforms. And as can be seen in Section 8.1, even a policy trained using an agent with a perfect linear response can be adapted to control physically simulated UAV platforms by changing the gain (action scales) applied to the output of the control policy.

### 6.3. Training and Using the Policy

The agent was trained on the environment detailed in Section 6.1 using the PPO2 algorithm implementation in the Stable Baselines project. Table 4 outlines the relevant training parameters used to produce the policy used in validation of the framework. The training was undertaken on a High End Desktop (HEDT), with a 32 Core (64 Thread) Processor (Threadripper 3970x) and took approximately five (5) hours to train. Training was split across sixty-four (64) parallel environments to improve the training time.

**Figure 7:**
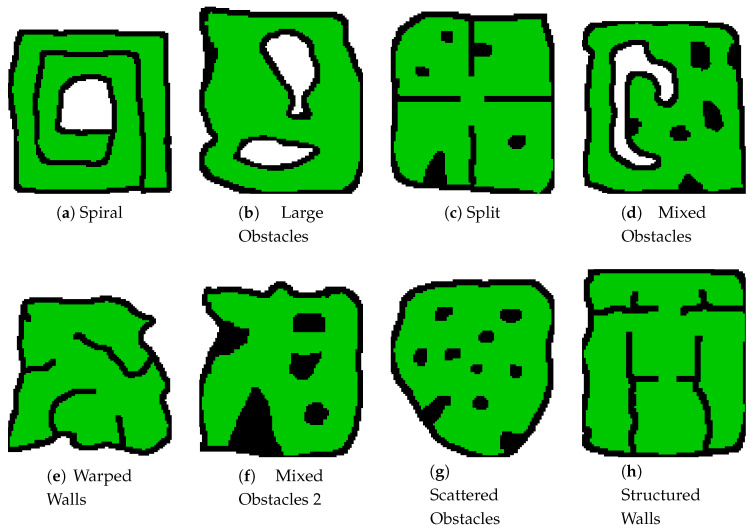
Training Maps One through Eight. **Black Cells**: Obstacle Cells, **Green Cells**: Traversable Cells.

Thirty two (32) different training maps were created and used to increase the domain randomisation of the training, with agents being randomly spawned into a safe location in one of those maps during each training run. Eight (8) of these training maps can be seen in Figure 7. By increasing the variety of features contained within these maps the control policy produced can be used on a variety of local search environments without the need for retraining, as is the case with the policy tested in this paper. Only abstraction of the environment and inference using the trained policy need to be conducted on-board the UAV for the local control stack, resulting in a very computationally efficient controller.

Furthermore, if a particular environment type is expected to be the only type of environment faced by the agents, that type of environment could be weighted more heavily in the training. For instance if the expected operation environments were only caves and tunnels, you would only need to train the policy on cave and tunnel style environments.

The policy naturally learns to avoid observed obstacles as collisions impart a penalty during training, and reduce the total reward by ending searches prematurely.

Use of the policy in the framework requires the local controller node to generate the correct observations for the control policy. If the agent is not in the desired target region, as dictated by the global planner, the local controller node considers both its current region and target region when generating observations. However, once the UAV transitions into its target region the controller node treats all other regions as obstacles during the local search. This prevents the UAV from exploring outside the designated target region until the region is searched and the global planner gives the agent another action.

The implementation of the local control policy also checks if the agent is about to enter a region occupied by another agent. If it does, the agent holds position until the region becomes clear. Combined with the global-planners cost penalty for navigating into spaces occupied by another agent, this ensures the agents are never within the local space of each other, preventing any inter-agent collisions.

## 7. Software Architecture

**Figure 8:**
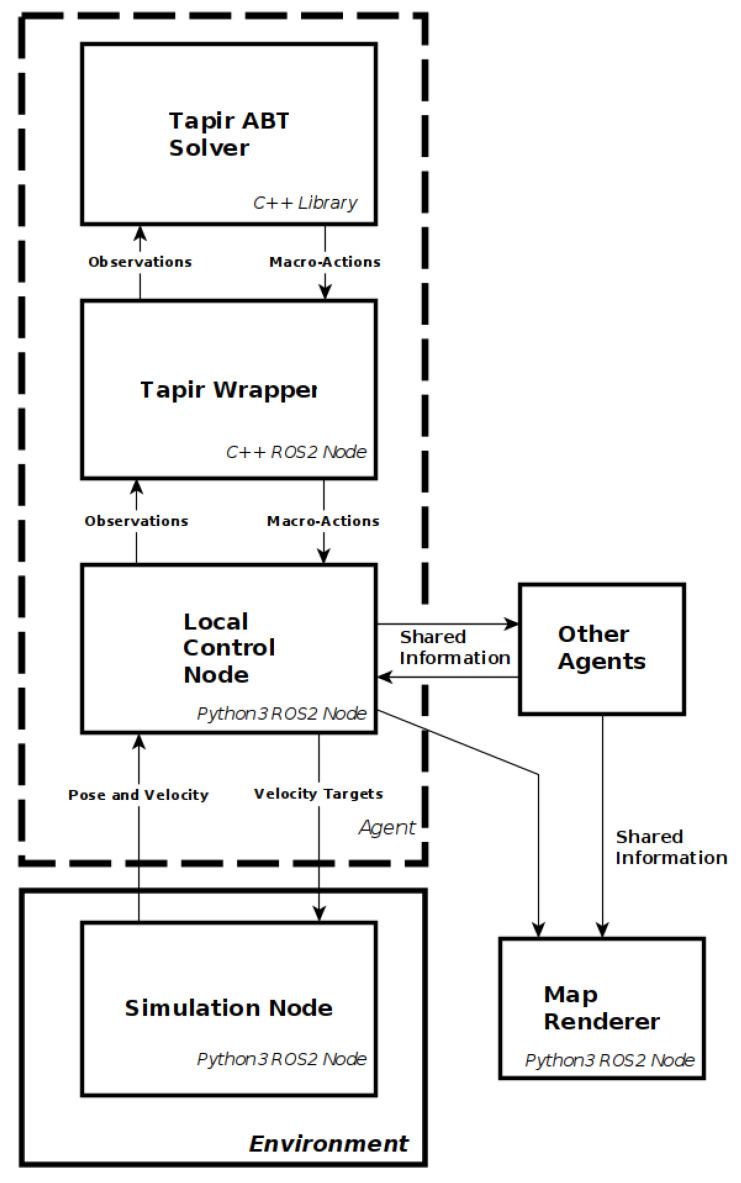
Software Architecture.

The components of the Framework were combined through the use of the Robotic Operation System 2 (ROS2) and executed on an Ubuntu 18.04 system. The software breakdown for an agent can be seen in Figure 8. The local control policy and global planner required the use of the stable-baselines python package and TAPIR ABT Implementation respectively, with custom models required for each. The full framework, along with a setup guide, is available at the following link: https://github.com/OryWalker/Multi-Agent-Target-Finding.

## 8. Experimental Results

Framework validation occured in two stages. First the performance of the Local Controller was confirmed. Once it was shown that the Local Controller was capable of controlling both ideal and physically simulated agents in an unseen environment, the full framework was tested using a varying number of ideal simulated agents on multi-region environments to validate overall performance. The following sections outline the testing undertaken.

### 8.1. Testing the Local Controller

**Figure 9:**
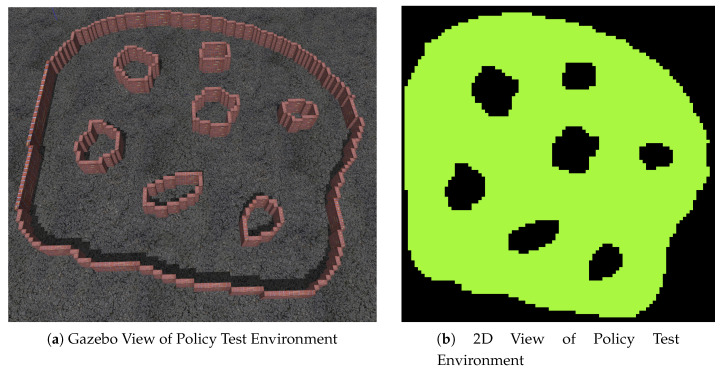
Policy Test Environment.

The local control policy and local controller were validated across three separate UAV platforms; the generic simulated agent that the policy was trained on as a baseline, and two physically simulated UAV platforms within the Gazebo simulation environment, the 3DR Iris and 3DR Solo. All agents were required to search the test environment show in Figure 9 to ninety-five (95) percent completion. The flyable area of the test environment was approximately one thousand five hundred (1500) square meters and the test environment was not part of the training environments. The baseline test using the default action scales ([0.25, 0.125]) and ideal simulated agent was run ten (10) times to obtain an idea of the time to search the environment. The IRIS and SOLO agents were then tested using a variety of action scales, with a total five (5) runs per configuration. The time to crash or finish the test was recorded. Table 5 shows the average times for the tests undertaken. If the agent crashed for the majority of the tests the time to crash is listed, while if the agent finished the test the majority of the time, the average time to finish is shown. For each test the number of crashes is listed, with five (5) meaning all runs failed for the physically simulated configurations.

It can be seen that the performance at the default action scales was undesirable. This is due to the sluggishness of the response of the IRIS and SOLO platforms when compared to the trained ideal agent. Increasing the action scale in increments of 0.125 for both the linear and angular action scales up to a maximum of 1 shows that the performance increases such that the physically simulated agents perform almost on par with the ideal agent the policy was trained on.

**Figure 10:**
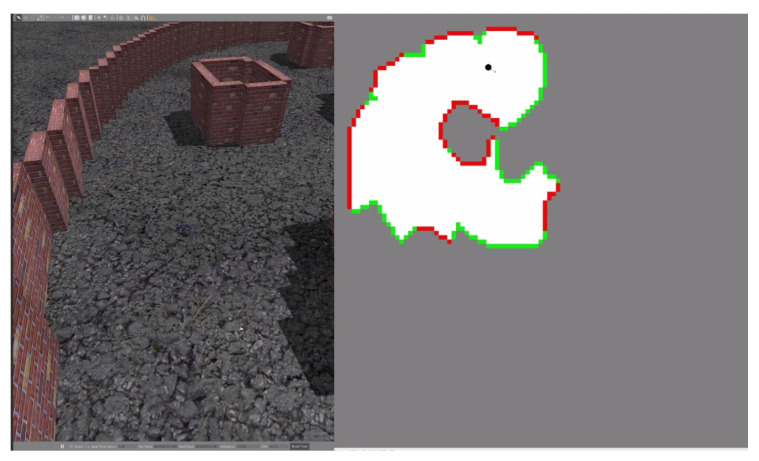
3DR Solo Policy Test Snapshot.

Figure 10 shows a snapshot of a 3DR Solo test using action scales of one (1.0). The full video of that test can be found at https://youtu.be/u2I5xYWlPuM.

### 8.2. Testing the Full Framework

**Figure 11:**
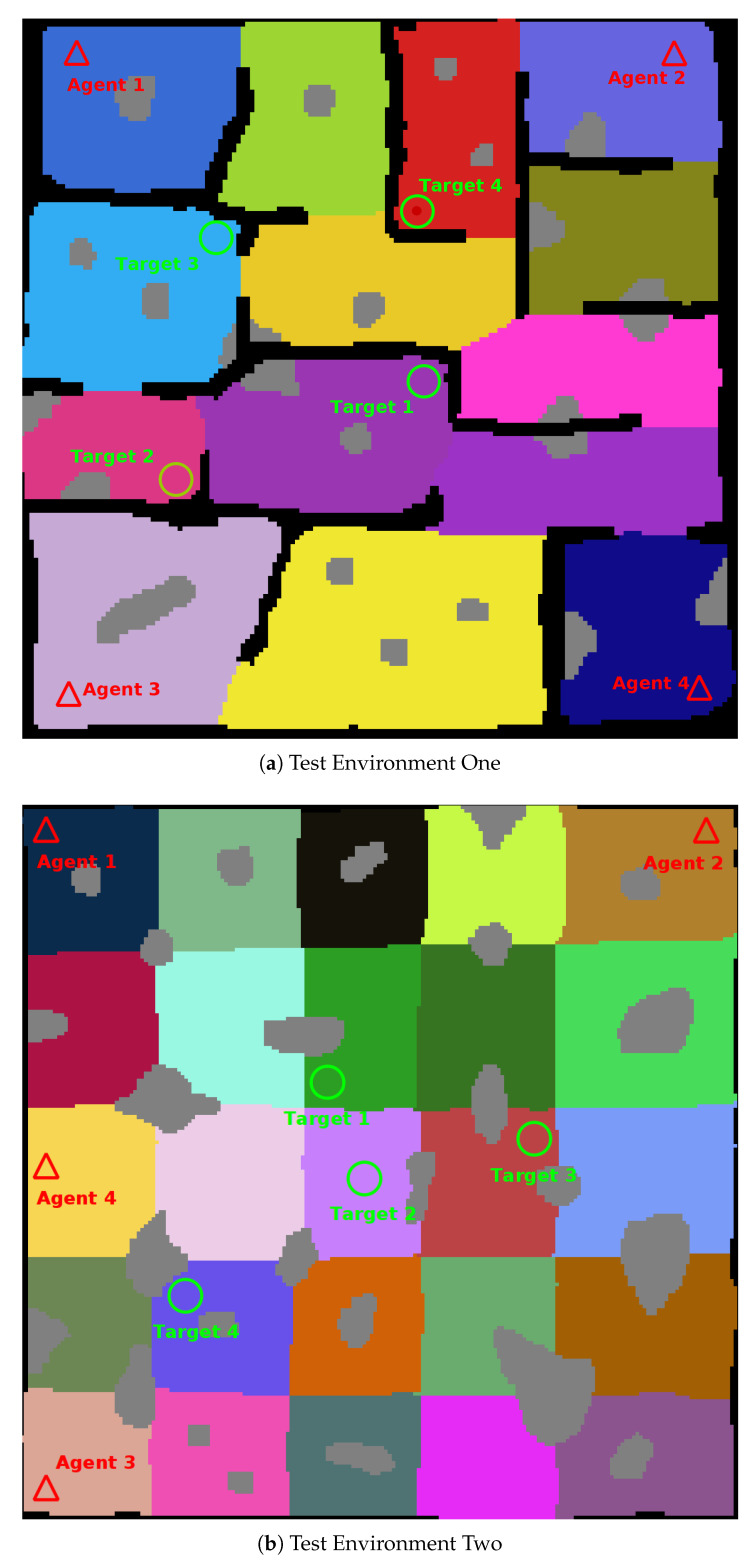
Complete Test Environments. **Black Cells**: Known Obstacle Cells, **White Cells**: Explored Empty Space Cells, **Coloured Cells**: Unexplored Empty Cells (Search Regions), **Grey Cells**: Unknown Obstacle Cells, **Triangles**: Agent Spawn Locations, **Circles**: Target Spawn Locations.

The combined performance of the framework was validated with generic simulated agents searching two test maps; a medium sized, fourteen (14) region environment, and a large twenty-five (25) region environment. Figure 11 shows both of the complete environments used for testing.

Testing was conducted using a variety of agent and target configurations. Test environment one was tested using one (1), two (2), three (3), and four (4) agent configurations. For each agent configuration five (5) tests were run for four (4) target locations, for a total of fifteen (20) test configurations for each agent configuration and a total of eighty (80) test runs for the first test environment.

The second test environment was also run eighty (80) times, using one (1), two (2), three (3), and four (4) agent configurations. No collisions with the environment were recorded for any of the test runs.

**Figure 12:**
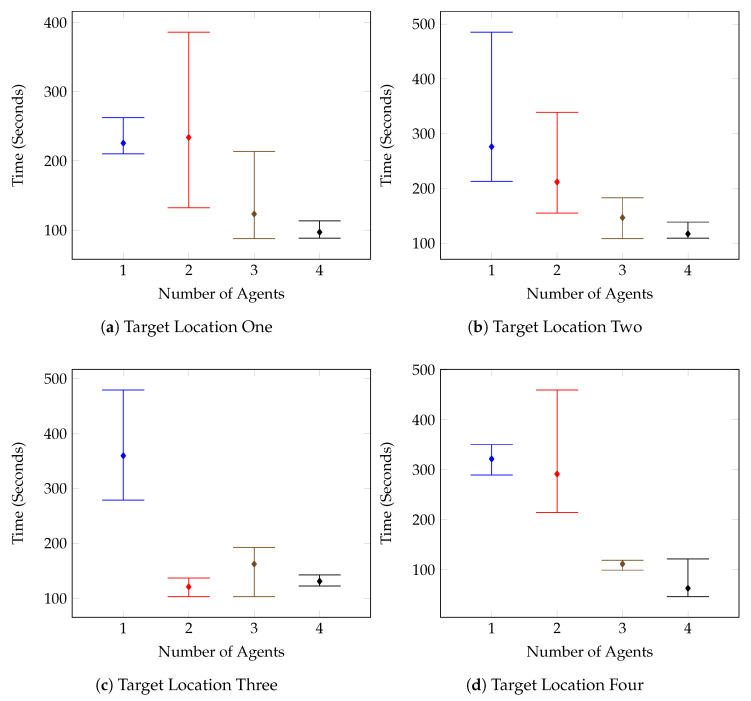
Number of Agents vs. Maximum, Mean and Minimum Times (Seconds)-Test Environment One.

**Figure 13:**
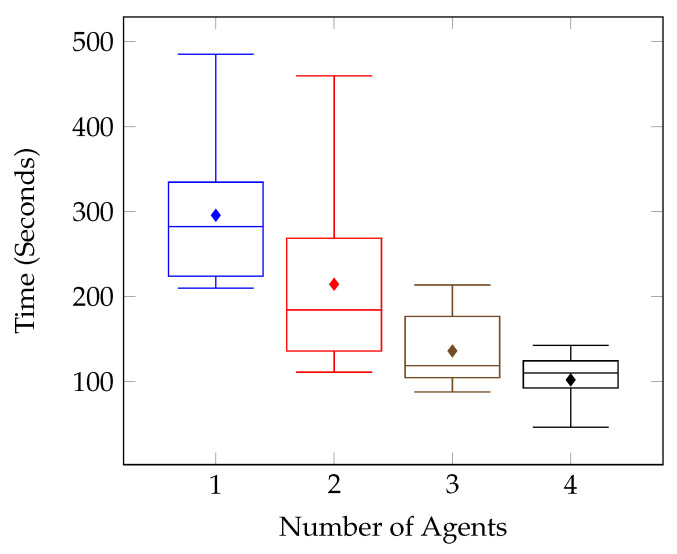
Number of Agents vs. Time (Seconds)-All Target Locations in Test Environment One.

**Figure 14:**
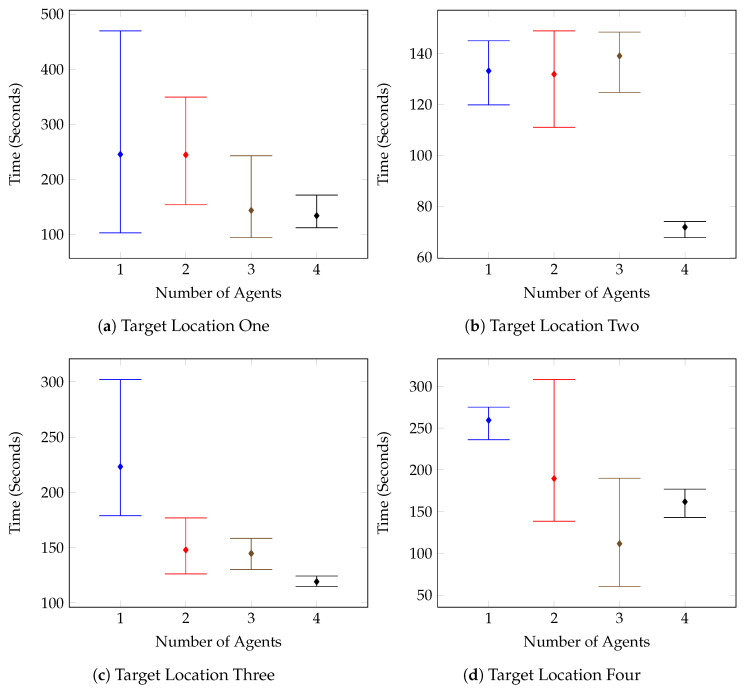
Number of Agents vs. Maximum, Mean and Minimum Times (Seconds)-Test Environment Two.

**Figure 15:**
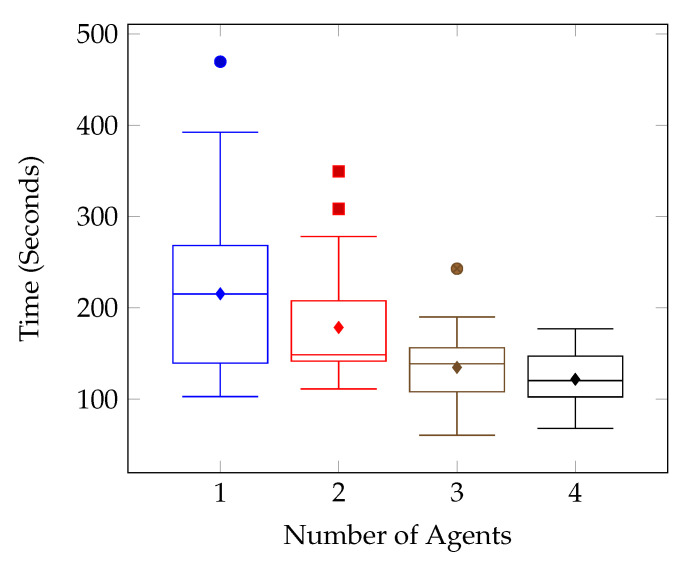
Number of Agents vs. Time (Seconds)-All Target Locations in Test Environment Two.

The numerical results can be seen notated in Table 6 and visualised in Figure 12, Figure 13, Figure 14 and Figure 15.

Figure 12 outlines the individual target tests for the first environment, and it can be seen that on the whole, the times improved as the number of agents increased. This is further supported by the combined results shown in Figure 13. There are however a few anomolous results such as in Figure 12c, which displays the results for the Test Environment One, Target Location Three tests. After increasing the number of agents to two, the test results remain mostly consistent, with the system performing the same with two agents as with three, and four.

**Figure 16:**
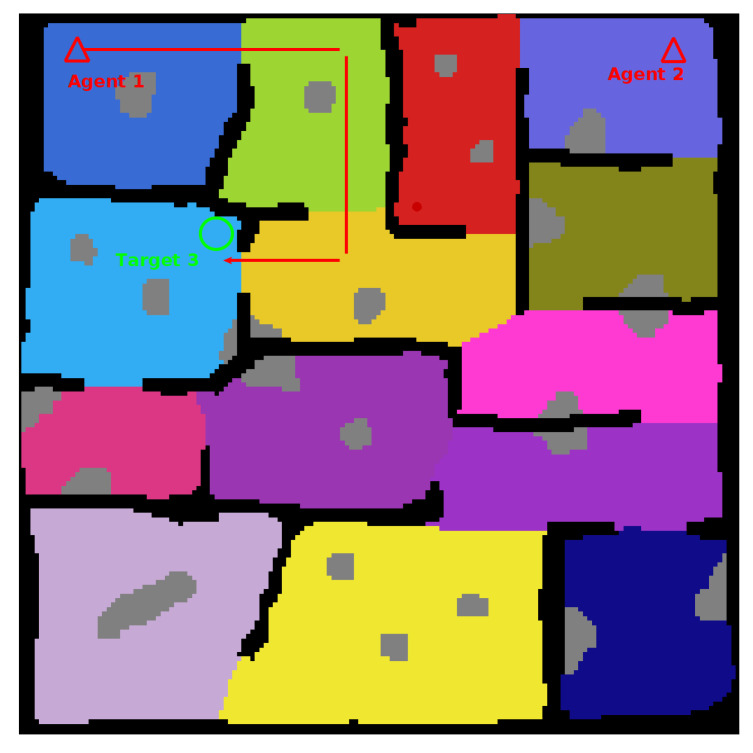
Target Three Test Example.

This is a result of the target location and the spawn of the second agent. The spawn of the second agent causes the first agent to consistently route almost directly toward the target location in an attempt to avoid planning conflicts. The route followed can be seen in Figure 16. These kinds of routing changes when adding agents are also responsible for other test case anomolies. Such as the Target Location Two test for Environment Two, shown in Figure 14b. In this case, it isn’t until the fourth agent is added that the agents route themselves in such a way that the target is found in half the time compared with the previous tests.

It can be seen from the test results that there was a general trend of improved search time and consistency as the number of agents increased.

**Figure 17:**
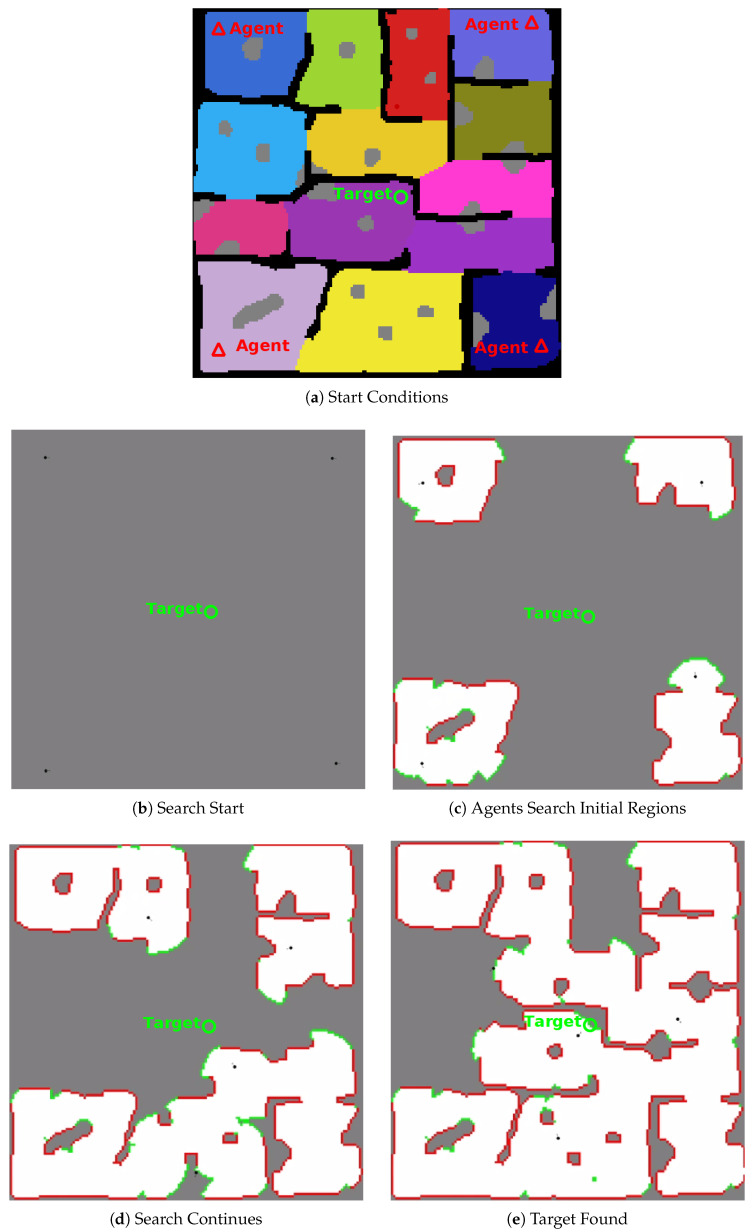
Four Agent Search of Test Environment One. **Grey Cells**: Unexplored Area, **Red Cells**: Observed Obstacle Cells, **Green Cells**: Safe Edges of Unexplored Region, **White Dotted Circle**: Target Location.

Figure 17 shows the progress and completion of a four (4) agent test on test environment one (1). The video at https://youtu.be/jh0dn33Ji0k shows a four (4) agent search of test environment one (1) at two (2) times playback speed.

## 9. Conclusions

This paper has shown that using the proposed local control training environment, a small two layer neural net is capable of learning to control a generic UAV agent to explore a two dimensional (2D) occupancy-map of an environment while avoiding previously unknown obstacles. It has also shown that such a policy can be extended from use with the trained generic simulated agent to two different physically simulated platforms (3DR Iris, and 3DR Solo) simply by changing the gain for the control policy output.

Furthermore, the completed framework detailed by this paper has been shown to enable simulated UAV agents to search arbitrarily shaped, GPS-denied and partially observable environments using the combination of POMDP based planning and Deep Reinforcement Learning based control, under the assumption of accurate SLAM.

Given the performance of the physically simulated UAVs within Gazebo, using the PX4 software stack, the framework in its current form could be applied to a real-world agent with an accurate SLAM system and a map type that could be converted to the necessary 2D grid style environment that this framework uses. However, future work aims to integrate the use of existing map types such as the octo-map format, 3-D environments, and to model imperfect SLAM and positional noise within the control environment. This would be done in an attempt to increase the applicability of the framework and reduce the work required to enable this framework on a variety of platforms.

While the current framework prevents agents from interacting in a local environment, producing a control policy that enables inter-agent collision avoidance and cooperation at a local level is a target for future work. Additionally, while this paper does not consider strategies for optimizing the swarm configuration, this is also a goal for future works. Finally other future work could also include: improving the global planner to respond to large changes in map structure and lack of prior information, integration and validation of the framework on real-world hardware and in real-world environments, and development of additional problem definitions such as point of interest surveying and multi-target finding and pursuit.

## Figures and Tables

**Table 1:** Global Planner POMDP Formulation.

**State-Space (S)**	UAV Locations
	Target Location
**Observations (O)**	Location of UAV Agents
	Target Seen Status for each Agent
**Actions (A)**	Move to a Connected Node
**Rewards (R)**	Cost for each step
	Cost for occupying same node as other agent
	Reward when the target is found

**Table 2:** Local Control Environment Formulation.

**State-Space (S)**	UAV Pose
	UAV Velocity
	Environment Map
**Observations (O)**	Map Observations
	Agent Velocities (x, y, yaw)
**Actions (A)**	Change velocity in any of the operational axes (x, y, yaw)
**Rewards (R)**	Reward for each cell explored
	Cost for collision equal to sum of cumulative reward

**Table 3:** Generic Simulated Agent Definition.

**Radius (m)**	0.5 metres
**Linear Velocity Limit (m/s)**	1.5
**Angular Velocity Limit (ω/s)**	π/4
**Response Type**	Linear
**Linear Action Scale (m/s)**	0.25
**Angular Action Scale (ω/s)**	0.125
**Sensor Type**	Front Facing Camera
**Sensor Range (m)**	7
**Sensor FOV (${}^{\circ }$ Horizontal)**	90

**Table 4:** Training Parameters.

**Policy**	PPO2-MLP
**Layers**	2
**Neurons per Layer**	64
**Normalised**	Yes
**Environments**	64
**Mini Batches**	32
**Learning Rate**	0.0002
**Gamma**	0.99
**Clip Range**	0.2
**NoPtEpochs**	10
**Lam**	0.925
**Max Grad Norm**	0.5
**Ent Coef**	0.0
**Steps**	1800

**Table 5:** Local Controller Test Results.

Action Scales	Average Time (sec)	Outcome	No. of Crashes
**Ideal Agent**			
**0.25 // 0.125**	127.62	**FINISH**	0
**3DR Solo**			
**0.25 // 0.125**	144.27	**CRASH**	5
**0.25 // 0.25**	126.52	**CRASH**	4
**0.375 // 0.375**	133.65	**CRASH**	4
**0.5 // 0.5**	163.42	**FINISH**	2
**0.625 // 0.625**	153.94	**FINISH**	0
**0.75 // 0.75**	171.86	**FINISH**	0
**0.875 // 0.875**	161.52	**FINISH**	0
**1.0 // 1.0**	145.08	**FINISH**	0
**3DR Iris**			
**0.25 // 0.125**	71.04	**CRASH**	5
**0.25 // 0.25**	45.83	**CRASH**	4
**0.375 // 0.375**	69.28	**CRASH**	4
**0.5 // 0.5**	153.33	**FINISH**	1
**0.625 // 0.625**	105.15	**FINISH**	4
**0.75 // 0.75**	122.82	**FINISH**	0
**0.875 // 0.875**	140.95	**FINISH**	1
**1.0 // 1.0**	138.32	**FINISH**	0

**Table 6:** Framework Test Results.

**Test Environment One**				
**Agents**	**One**	**Two**	**Three**	**Four**
**Min Time (Sec)**	210.07	103.08	87.64	46.05
**Max Time (Sec)**	485.31	459.75	213.66	142.61
**Mean Time (Sec)**	295.70	214.46	135.96	101.92
**Test Environment Two**				
**Agents**	**One**	**Two**	**Three**	**Four**
**Min Time (Sec)**	102.70	110.98	60.28	67.67
**Max Time (Sec)**	469.45	349.25	242.69	176.83
**Mean Time (Sec)**	215.22	178.34	134.61	121.60

## Abbreviations

The following abbreviations are used in this manuscript:

MDP	Markov Decision Process
POMDP	Partially Observable Markov Decision Process
UAV	Unmanned Aerial Vehicle
ABT	Adaptive Belief Tree
PPO	Proximal Policy Optimisation
